# Memantine, an Antagonist of the NMDA Glutamate Receptor, Affects Cell Proliferation, Differentiation and the Intracellular Cycle and Induces Apoptosis in *Trypanosoma cruzi*

**DOI:** 10.1371/journal.pntd.0002717

**Published:** 2014-02-27

**Authors:** Flávia Silva Damasceno, María Julia Barisón, Elisabeth Mieko Furusho Pral, Lisvane Silva Paes, Ariel Mariano Silber

**Affiliations:** Unit for Drug Discovery, Departamento de Parasitologia, Instituto de Ciências Biomédicas, Universidade de São Paulo, São Paulo, Brazil; Federal University of São Paulo, Brazil

## Abstract

Chagas' disease is caused by the protozoan parasite *Trypanosoma cruzi* and affects approximately 10 million people in endemic areas of Mexico and Central and South America. Currently available chemotherapies are limited to two compounds: Nifurtimox and Benznidazole. Both drugs reduce the symptoms of the disease and mortality among infected individuals when used during the acute phase, but their efficacy during the chronic phase (during which the majority of cases are diagnosed) remains controversial. Moreover, these drugs have several side effects. The aim of this study was to evaluate the effect of Memantine, an antagonist of the glutamate receptor in the CNS of mammals, on the life cycle of *T. cruzi*. Memantine exhibited a trypanocidal effect, inhibiting the proliferation of epimastigotes (IC_50_ 172.6 µM). Furthermore, this compound interfered with metacyclogenesis (approximately 30% reduction) and affected the energy metabolism of the parasite. In addition, Memantine triggered mechanisms that led to the apoptosis-like cell death of epimastigotes, with extracellular exposure of phosphatidylserine, increased production of reactive oxygen species, decreased ATP levels, increased intracellular Ca^2+^ and morphological changes. Moreover, Memantine interfered with the intracellular cycle of the parasite, specifically the amastigote stage (IC_50_ 31 µM). Interestingly, the stages of the parasite life cycle that require more energy (epimastigote and amastigote) were more affected as were the processes of differentiation and cell invasion.

## Author Summary

*Trypanosoma cruzi* is a parasite transmitted to mammal hosts by insect vectors known as kissing bugs. This species can result pathogenic for humans, causing Chagas' disease in the Americas. Its treatment relies on two drugs discovered more than 40 years ago. Besides their toxicity, a main drawback of these drugs is the fact that they are highly efficient only during the acute phase of the infection. But due to the lack of specific symptoms, the acute phase of the infection is largely not diagnosed. In fact, most of patients are diagnosed in the chronic phase, where the treatments are not satisfactory. In view of that, it is urgent to look for new drugs with low toxicity and able to kill the parasite in chronic patients. On the basis of previous finding, we looked for drugs against glutamate recognizing surface molecules, keeping special attention on those that are already in use in humans for other purposes (this strategy is called drug repositioning, and allow to save time and money in clinical trials: several parameters such as toxicity, pharmacokinetics, side effects in humans are already known). Here we report that Memantine, a NMDA glutamate receptors antagonist already in use to treat Alzheimer's disease, presents interesting perspectives as a trypanocidal drug.

## Introduction

*Trypanosoma cruzi* is the etiological agent of Chagas' disease, which affects approximately 10 million people living in endemic areas of Mexico and Central and South America, with 28 million people at risk of infection [1]. *T. cruzi* has a complex life cycle that alternates between a reduviid insect vector and mammalian hosts (humans among them). During its biological cycle, the parasite differentiates several times between infective, non-dividing forms and dividing forms that inefficiently or are unable to infect mammalian cells. Epimastigotes, the replicative form in the insect vector, colonize the digestive tract and differentiate into metacyclic trypomastigotes, the insect-derived infective form, in the terminal midgut. During a blood meal on a mammalian host, the insects defecate and deposit these forms with the feces, which are internalized by the mammalian host and invade cells where they differentiate into the replicative amastigote stage in the cytoplasm. Amastigotes replicate by binary fission until differentiating into mammal-derived trypomastigotes, passing through a transient epimastigote-like stage [2], [3]. These trypomastigotes induce the lysis of the host cells, bursting into the extracellular milieu where they invade new cells or reach the bloodstream. The parasites disseminate throughout the infected mammal through the blood and can eventually be taken up by a new reduviid insect during a blood meal. In the midgut, the ingested trypomastigotes differentiate into epimastigotes, which replicate, thereby colonizing a new insect vector [3].

The clinical evolution of Chagas' disease in humans can be divided into two phases: acute and chronic. The acute phase is usually asymptomatic with patent parasitemia and non-specific symptoms. The chronic phase is characterized by infrequent tissue parasitism and subpatent parasitemia that persists for the life of the host. Most patients in the chronic phase (60–70%) will never develop clinically apparent disease. However, approximately 30–40% of chronic patients will develop important physiological alterations: the heart is affected, with hypertrophy and dilatation, and furthermore, the digestive tract, mainly the esophagus and large intestine, are affected, with dilatation and the appearance of megaviscera [4]–[6] as reviewed in reference [7].

Chemotherapy relies on two drugs that were discovered approximately 40 years ago: Nifurtimox and Benznidazole. Both drugs are effective for treating the acute phase of the disease. However, their efficacy in treating the chronic phase, when most patients are diagnosed, is controversial [7]. Moreover, drawbacks for both drugs have been reported, such as serious toxic side effects and more recently, the emergence of drug-resistant parasites. These facts underscore the urgent need to intensify the search for new drugs against *T. cruzi* [7], [8].

Our group has been exploring drug repositioning strategies, which are being widely employed for the discovery of novel therapeutic strategies to treat tropical diseases [9], [10]. This strategy seeks new uses for drugs that are already approved for the treatment of diseases in humans. Paveto and colleagues have suggested that *T. cruzi* epimastigotes have an N-methyl-D-aspartate (NMDA)-type L-glutamate receptor that is involved in the control of cytosolic Ca^2+^ levels, functionally analogous to that reported in neural cells [11]. Moreover, our group characterized a glutamate transporter [12] which is able to bind NMDA, behaving as a glutamate receptor (unpublished data). In addition, analogs of amantadine and Memantine (1,2,3,5,6,7-hexahydro-1,5:3,7-dimethano-4-benzoxonin-3-yl)amines with NMDA receptor antagonist activity were also demonstrated to have significant trypanocidal activity against *Trypanosoma brucei* [13]. These data led us to hypothesize that trypanocidal activities are present in compounds directed against mammalian glutamate receptors. In the present work, we tested the anti-*T. cruzi* activity of three compounds that have antagonistic effects on NMDA receptors: Amantadine and Memantine, tricyclic amines with low-to-moderate affinity for the NMDA receptor and used for the treatment of Alzheimer's disease [14], and MK-801, which is currently being tested in preclinical studies [15]. Memantine, an uncompetitive blocker of continuously overactivated NMDA receptors in neurons, exhibited the highest antiproliferative activity on epimastigotes and a relevant trypanocidal effect against infective forms of *T. cruzi*. Our experiments show that Memantine mobilizes intracellular Ca^2+^ and induces apoptosis, which supports the presence of a receptor with similar activity to glutamate NMDA receptors that can be used as drug targets against this parasite.

## Materials and Methods

### Reagents

Memantine was purchased from TOCRIS; MK-801, MTT (3-(4,5-dimethylthiazol-2-yl)-2,5-diphenyltretazolium bromide) and a kit for bioluminescent somatic cells were purchased from Sigma-Aldrich (St. Louis, MO, USA). Amplex red, horseradish peroxidase, Fluo-4 AM and annexin V-FITC were purchased from Invitrogen (Eugene, Oregon, USA). Culture medium and fetal calf serum (FCS) were purchased from Cultilab (Campinas, SP, Brazil).

### Cells and parasites

The Chinese Hamster Ovary cell line (CHO-K_1_) was cultivated in RPMI medium supplemented with 10% heat-inactivated FCS, 0.15% (w/v) NaCO_3_, 100 units mL^−1^ penicillin and 100 µg mL^−1^ streptomycin at 37°C in a humidified atmosphere containing 5% CO_2_. *T. cruzi* CL strain clone 14 epimastigotes [16] were maintained in the exponential growth phase by subculturing every 48 h in Liver Infusion Tryptose (LIT) medium supplemented with 10% FCS at 28°C. Trypomastigotes were obtained by infection of CHO-K_1_ cells with trypomastigotes as described previously [17]. Trypomastigotes were collected from the extracellular medium five or six days after infection.

### Growth inhibition assays

*T. cruzi* epimastigotes in the exponential growth phase (5.0–6.0×10^7^ cells mL^−1^) were cultured in fresh LIT medium. The cells were treated with different concentrations of drugs or not treated (negative control). A combination of Rotenone (60 µM) and Antimycin (0.5 µM) was used as a positive control for inhibition as previously described [5]. The cells (2.5×10^6^ mL^−1^) were transferred to 96-well culture plates and incubated at 28°C. Cell proliferation was quantified by reading the optical density (OD) at 620 nm for eight days. The OD was converted to cell density values (cells per mL) using a linear regression equation previously obtained under the same conditions. The concentration of compounds that inhibited 50% of parasite proliferation (IC_50_) was determined during the exponential growth phase (five days) by fitting the data to a typical sigmoidal dose-response curve using OriginPro8. The compounds were evaluated in quadruplicate in each experiment. Except where otherwise indicated, for experimental purposes epimastigotes (1.0×10^6^ cells mL^−1^) were cultured in LIT and treated with a concentration corresponding to the IC_50_ (172.6 µM) Memantine or not treated (control). Before conducting the experiments, epimastigotes were washed twice in PBS and resuspended in 50 µl of binding buffer (10 mM HEPES, 140 mM NaCl and 2.5 mM CaCl_2_, pH 7.4). The results shown here correspond to three independent experiments.

### Analysis of extracellular phosphatidylserine exposure

Parasites were treated with Memantine for four days or not treated (control). Annexin V-FITC and propidium iodide were added to the final concentration indicated by the manufacturer. The cells were analyzed by flow cytometry on a Guava cytometer (General Electric).

### Hydrogen peroxide production

Epimastigote were treated with Memantine for 24 hours, washed and resuspended in PBS (5 mM succinate). The cells were incubated with 12 µM amplex red and 0.05 U mL^−1^ horseradish peroxidase. Fluorescence was monitored at a λ_excitation_ of 563 nm and a λ_emission_ of 587 nm on a Spectra Max M3 fluorometer (Molecular Devices). Calibration was performed using hydrogen peroxide as a standard.

### Analysis of intracellular Ca^2+^ levels

Parasites (1.0×10^8^ cells) treated Memantine for four days were incubated with 5 µM Fluo-4 AM (Invitrogen) for one hour at 28°C. After this period, the cells were washed twice with HEPES-glucose (50 mM HEPES, 116 mM NaCl, 5.4 mM KCl, 0.8 mM MgSO_4_, 5.5 mM glucose and 2 mM CaCl_2_, pH 7.4), resuspended in the same buffer and distributed into 96-well plates (2.5×10^7^ per well) in triplicate. Readings were performed on a Spectra Max M3 fluorometer using a λ_excitation_ of 490 nm and a λ_emission_ of 518 nm [18].

### Determination of *T. cruzi* intracellular ATP levels

Intracellular ATP levels were measured in treated (or not) epimastigote forms. To assess the effect of Memantine on the levels of intracellular ATP, a kit for bioluminescent somatic cells purchased from Sigma-Aldrich was used according to the manufacturer's instructions. Briefly, 50 µl of PBS was added to 100 µl of cellular ATP-releasing reagent and added to 50 µl of parasite suspension containing 5.0×10^6^ treated or untreated (control) cells mL^−1^. The concentration of ATP was determined using a standard curve of different concentrations of ATP. Luminescence was obtained by the reaction between luciferase and the ATP that was released after cell lysis. Light emission levels were measured on a Lumat LB 9507 luminometer at 570 nm.

### Effect of Memantine on metacyclogenesis

Epimastigotes (5.0×10^6^ cells mL^−1^) were grown in LIT medium, transferred to Grace's medium [19] and treated or not treated (control) with 172.6 µM Memantine (IC_50_ value). On the sixth day, after transfer, the parasites were counted in a Neubauer chamber, and the percentage of metacyclic forms was determined.

### Effect of Memantine on mammalian cell viability

CHO-K_1_ cells (5.0×10^5^ cells mL^−1^) were seeded in 24-well plates in RPMI medium supplemented with FCS (10%) with different concentrations of drugs or not treated (control). Cell viability was evaluated 48 h after the initiation of treatment using the MTT assay [20]. The IC_50_ was determined by fitting the data to a typical sigmoidal dose-response curve using OriginPro8.

### Effect of Memantine on trypomastigote invasion

CHO-K_1_ cells (5.0×10^4^ per well) were maintained in 24-well plates in RPMI medium supplemented with 10% FBS and maintained at 37°C. After 24 h, the cells were infected with trypomastigote forms (2.5×10^6^ per well) and treated with different concentrations of Memantine (50–300 µM) for four hours. After this period, free parasites and the Memantine were removed. The infected cells were washed twice with PBS. The RPMI medium was replaced, and the plates were incubated at 33°C. Trypomastigotes were collected from the extracellular medium on the fifth day and counted in a Neubauer chamber.

### Effect of Memantine on trypomastigote bursting

CHO-K_1_ cells (5.0×10^4^ per well) were maintained in 24-well plates in RPMI medium supplemented with 10% FBS and maintained at 37°C. After 24 h, the cells were infected with trypomastigote forms (2.5×10^6^ per well) for four hours. After this period, free parasites were removed. The infected cells were washed twice with PBS, the RPMI medium was replaced, and the cells were kept in culture in the presence of different concentrations of Memantine (5–300 µM). The plates were then incubated at 33°C. Trypomastigotes were collected from the extracellular medium on the fifth day and counted in a Neubauer chamber.

### Effect of Memantine on intracellular stages

CHO-K_1_ cells (5.0×10^4^ per well) were maintained in 24-well plates in RPMI medium supplemented with 10% FBS and incubated at 37°C. After 24 h, the cells were infected with trypomastigote forms (2.5×10^6^ per well) for four hours. The infected cells were washed twice with PBS, the RPMI medium was replaced, and the cells were treated at different times during invasion, after 24 h (amastigote stage) and after 60 h (epimastigote-like stage) with 31 µM Memantine (corresponding to the IC_50_ value obtained for the treatment of infected cells). The plates were incubated at 33°C. Trypomastigotes were collected from the extracellular medium on the fifth day and counted in a Neubauer chamber.

### Statistical analysis

One-way ANOVA followed by the Tukey post-test was used for statistical analysis. The *T* test was used to analyze differences between groups. P<0.05 was considered statistically significant.

## Results

### Memantine affects the growth of epimastigote forms

**Figure 1:**
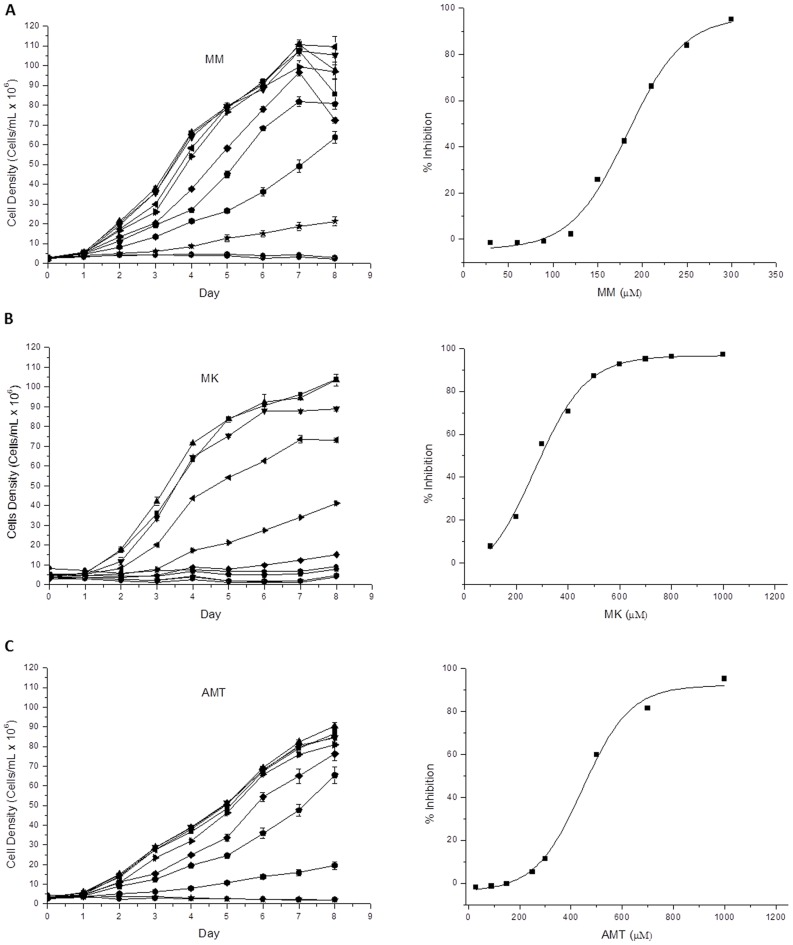
Growth curve of epimastigote forms of *Trypanosoma cruzi*. **Left:** growth curves in the presence of different concentrations of compounds. Treatments with (A) Memantine (MM), (B) MK-801, (C) Amantadine (AMT); at 28°C and 7.4 pH: black square: 0 µM; black up-pointing triangle: 30 µM (MM or AMT), 100 µM (MK); black down-pointing triangle: 60 µM (MM), 90 µM (AMT), 200 µM (MK); black left-pointing triangle: 90 µM (MM), 150 µM (AMT), 300 µM (MK); black right-pointing triangle: 120 µM (MM), 250 µM (AMT), 400 µM (MK); black diamond: 150 µM (MM), 400 µM (AMT), 500 µM (MK); black pentagon: 180 µM (MM), 500 µM (AMT), 600 µM (MK); black hexagon: 210 µM (MM), 700 µM (AMT or MK); black star: 250 µM (MM), 1,000 µM (AMT), 800 µM (MK); inverse white circle: 300 µM (MM), 1 mM (MK); black circle: Inhibition control (0.5 µM antimycin and 60 µM rotenone). **Right:** dose-response curves.

To investigate the possible presence of targets for mammalian NMDA glutamate receptor inhibitors, leading to a trypanocidal activity, Amantadine, Memantine and MK-801 were evaluated. A preliminary screening for the ability of these compounds to inhibit epimastigote growth was performed. *T. cruzi* epimastigotes were cultured in LIT medium with different concentrations of the selected drugs or no drug (control). Amantadine, Memantine and MK-801 produced a dose-dependent inhibition of epimastigote growth at 28°C and pH 7.5, the optimal growth conditions for these cells. The observed growth differences between the treated cells and the control were statistically significant (p<0.05), and the IC_50_ was determined to be 172.6 µM for Memantine, 300 µM for MK-801 and 451.2 µM for Amantadine (Figure 1A, 1B and 1C, respectively). In spite of being a relatively high IC_50_ when compared to that obtained herein for Benznidazole, (which resulted to be 7 µM, see Figure S1), the fact that Memantine is considered a safe drug for humans (few side effects have been reported) at relatively high doses (up to 20 mg/kg day), together with the facts that is commercially available and is inexpensive, led us to choice it for further study by investigating its effects on the biological processes of *T. cruzi*.

### Memantine leads to ROS production, transient increases in intracellular Ca^2+^ and programmed cell death (PCD) in epimastigote forms

**Figure 2:**
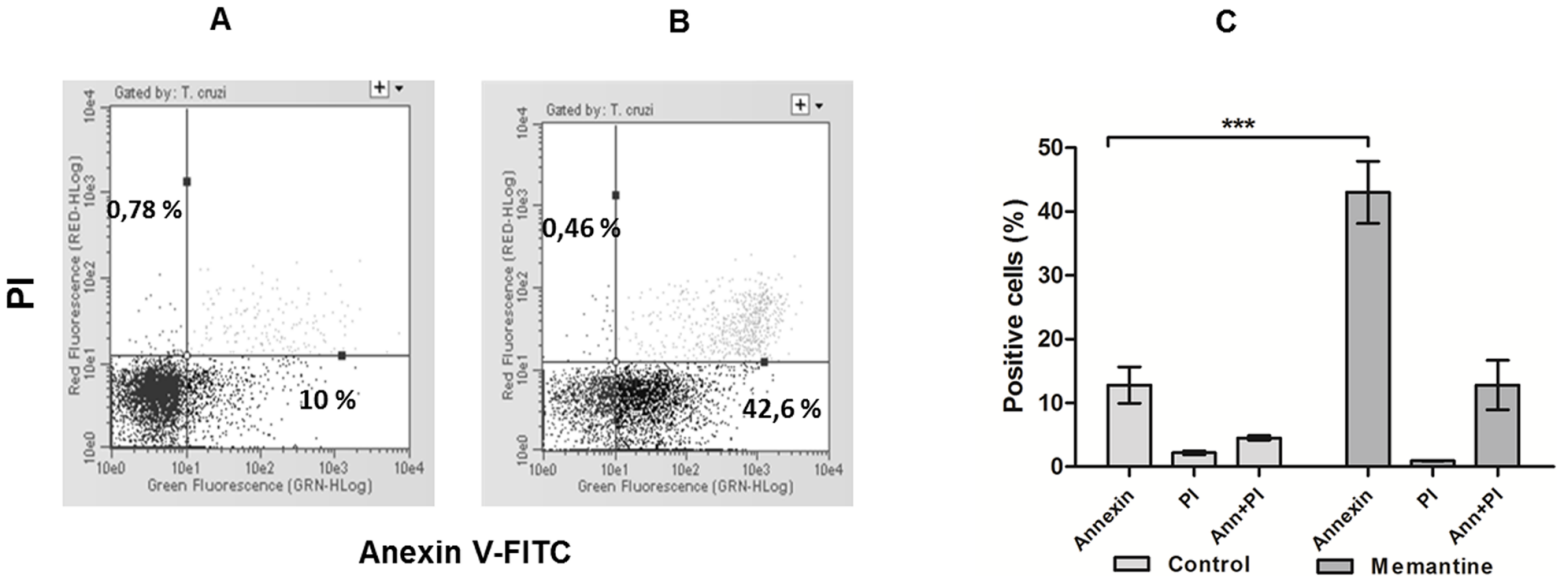
Analysis of extracellular phosphatidylserine exposure. Panel A: untreated parasites. Panel B: treated parasites. The parasites were treated with Memantine (172.6 µM) or not treated (control) for 4 days. After this period, the parasites were labeled with propidium iodide (PI) and annexin V and analyzed by flow cytometry. Panel C: quantitative analysis of three independent experiments (*T* test: ***: p<0.001).

**Figure 3:**
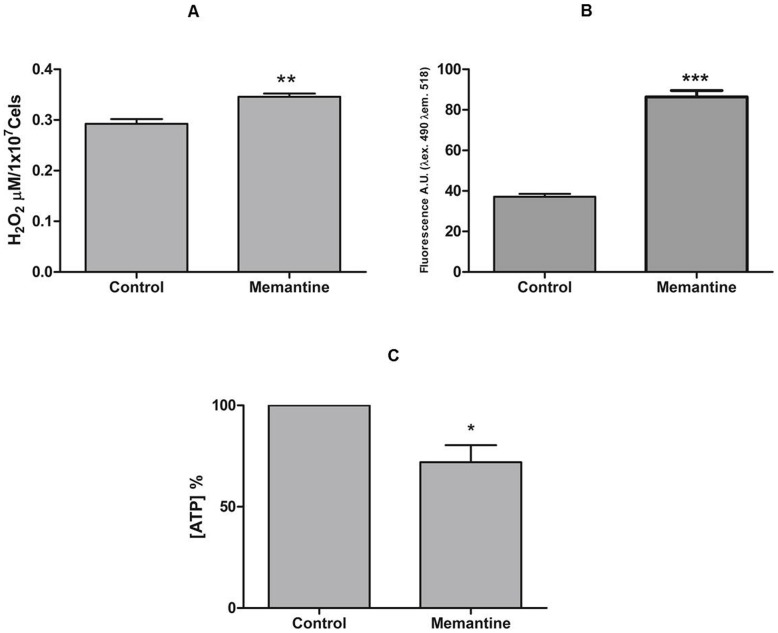
Quantification of H_2_O_2_, Ca^2+^ and ATP levels in *T. cruzi*. Panel A: **H_2_O_2_ levels**, parasites treated with Memantine (172.6 µM) or not treated (control) for 24 hours. After this period, the parasites (1.0×10^7^) were incubated with 25 µM amplex red, and 0.05 U mL^−1^ horseradish peroxidase and analyzed on a fluorometer (λ_ex_ 563 nm and λ_em_ 587 nm). Panel B: **Ca^2+^ levels**, parasites were treated for 4 days and incubated with Fluo-4 AM (5 µM) for 1 hour at 28°C, washed twice in HEPES-glucose and evaluated on a fluorometer (λ_ex_ 490 nm and λ_em_ 518 nm). Panel C: **ATP levels**, parasites were treated for 30 hours, and the levels of ATP were assessed using a bioluminescent assay kit (Sigma-Aldrich) and analyzed on a luminometer (λ 570 nm). *T* test: *: p<0.05; **: p<0.01; ***: p<0.001.

**Figure 4:**
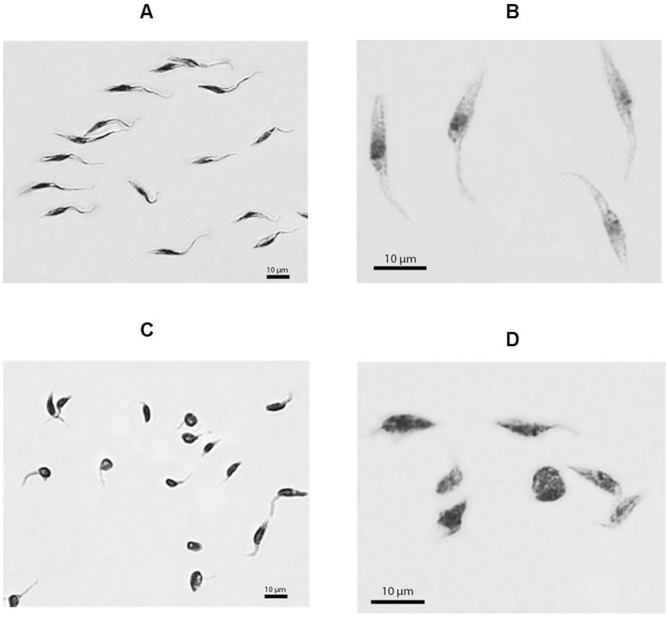
*Trypanosoma cruzi* epimastigote forms, 4^th^ day of growth. Panel A and B: untreated parasites. Panel C and D, parasites treated with Memantine (172.6 µM).

Programmed cell death is characterized by morphological and biochemical changes. A major change observed in cells undergoing PCD is exposure of phosphatidylserine on the extracellular face of the cytoplasmic membrane. Treated parasites were incubated with annexin V-FITC to assess external exposure of phosphatidylserine (feature of PCD) and propidium iodide to assess the possible rupture of the parasite membrane (feature of necrosis), and were further evaluated by flow cytometry. As shown (Figure 2A), untreated parasites (control) showed 10% positivity for phosphatidylserine exposure, whereas the parasites treated with Memantine showed 42% positivity (Figure 2B). Another type of necrotic process was excluded because the maintenance of parasite membrane integrity was confirmed by the absence of propidium iodide staining (Figure 2C). To confirm that Memantine induces apoptosis in epimastigotes, hallmarks for this process in trypanosomatids, such as an increase in reactive oxygen species (ROS), decreased ATP levels, increased intracellular Ca^2+^ levels and cell shrinkage [21]–[23], were explored. To evaluate the production of H_2_O_2_ in parasites treated with Memantine, epimastigote forms were treated with 172.6 µM Memantine (concentration corresponding to the IC_50_ value). After treatment for 24 hours, the parasites were incubated with amplex red and horseradish peroxidase. As observed, treated parasites produced a slightly increased amount of H_2_O_2_ than untreated parasites (Figure 3A). To determine intracellular concentrations of Ca^2+^, epimastigote forms were incubated with Memantine (172.6 µM) or no drug (control) for four days. After treatment, the parasites were incubated with Fluo-4 and analyzed by fluorometry. Treated parasites exhibited higher intracellular Ca^2+^ concentrations compared with untreated parasites (Figure 3B). The levels of intracellular ATP in treated and untreated cells were also determined using a bioluminescence assay. Intracellular ATP levels decreased in the treated parasites compared with the control (untreated parasites) (Figure 3C), indicating that the energy metabolism of the parasite is affected by the drug. Finally, we evaluated potential morphological changes in treated parasites compared with the control (Figure 4). Epimastigotes treated with Memantine exhibit dramatic changes in morphology (Figure 4C–D), presenting a characteristic rounded shape corresponding to shrinkage, a feature that is also described for apoptotic cells including trypanosomatids [23], [24]. These changes were reflected by changes on the values obtained for the forward and side light scattering (Table 1).

**Table 1:** Forward and side scattering values for epimastigotes treated or not with Memantine.

Treatment	1Forward Scatter	1Side Scatter
Control	64.08	107.77
Memantine	110.58	68.79

1 Geometrical mean of scatter values of epimastigotes treated or not with 172.6 µM Memantine.

### Effect of Memantine on metacyclogenesis

**Figure 5:**
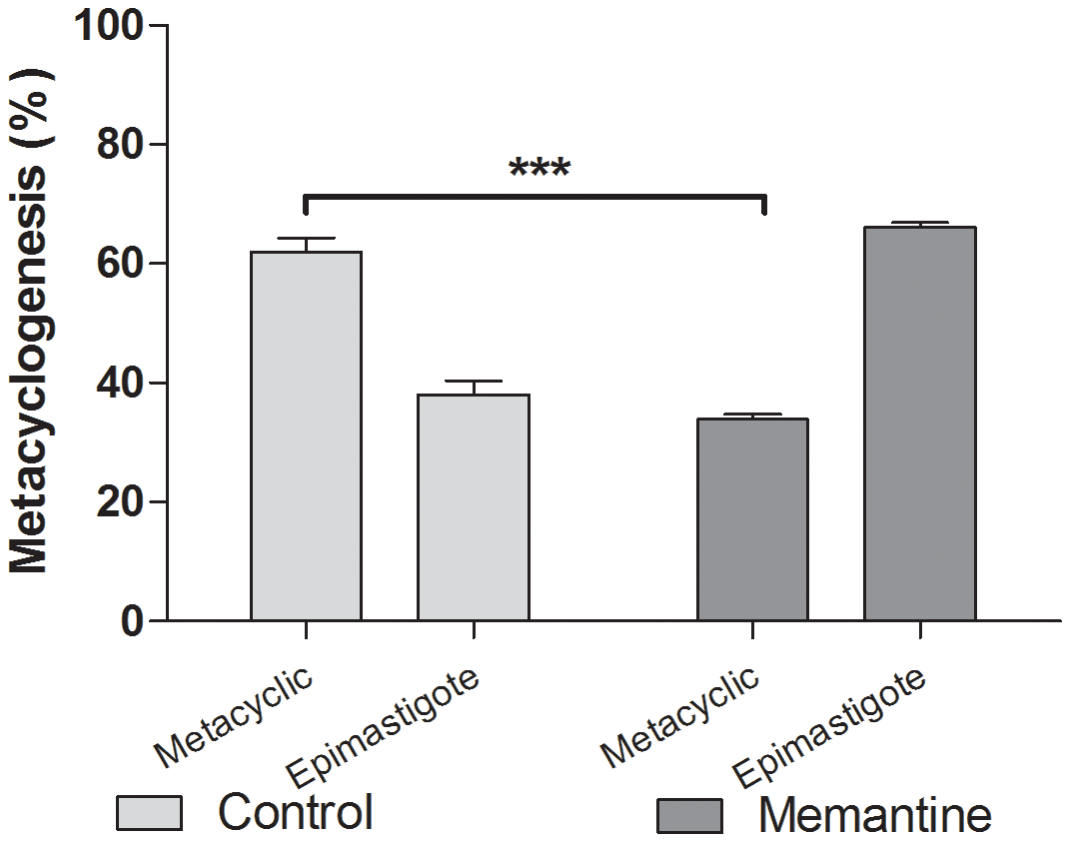
Effect of Memantine on metacyclogenesis. Epimastigote forms were grown in LIT medium, transferred to Grace's medium, and treated with Memantine (172.6 µM) or not treated. On the 9th day, the number of metacyclic forms was determined in a Neubauer chamber (*T* test: ***: p<0.001).

Because Memantine produced apoptotic activity in epimastigotes, we evaluated whether the drug could interfere with parasite differentiation. Metacyclogenesis is a well-characterized process in *T. cruzi* that involves transient modulation of Ca^2+^ levels and is dependent upon the parasite's metabolic status [25], both of which were affected by Memantine. On this basis, we evaluated the effect of Memantine on metacyclogenesis. Memantine-treated parasites sustained a 30% decrease in the number of metacyclic forms compared with the control (parasites without treatment) (Figure 5).

### Effect of Memantine on invasion and the intracellular cycle of *T. cruzi*

**Figure 6:**
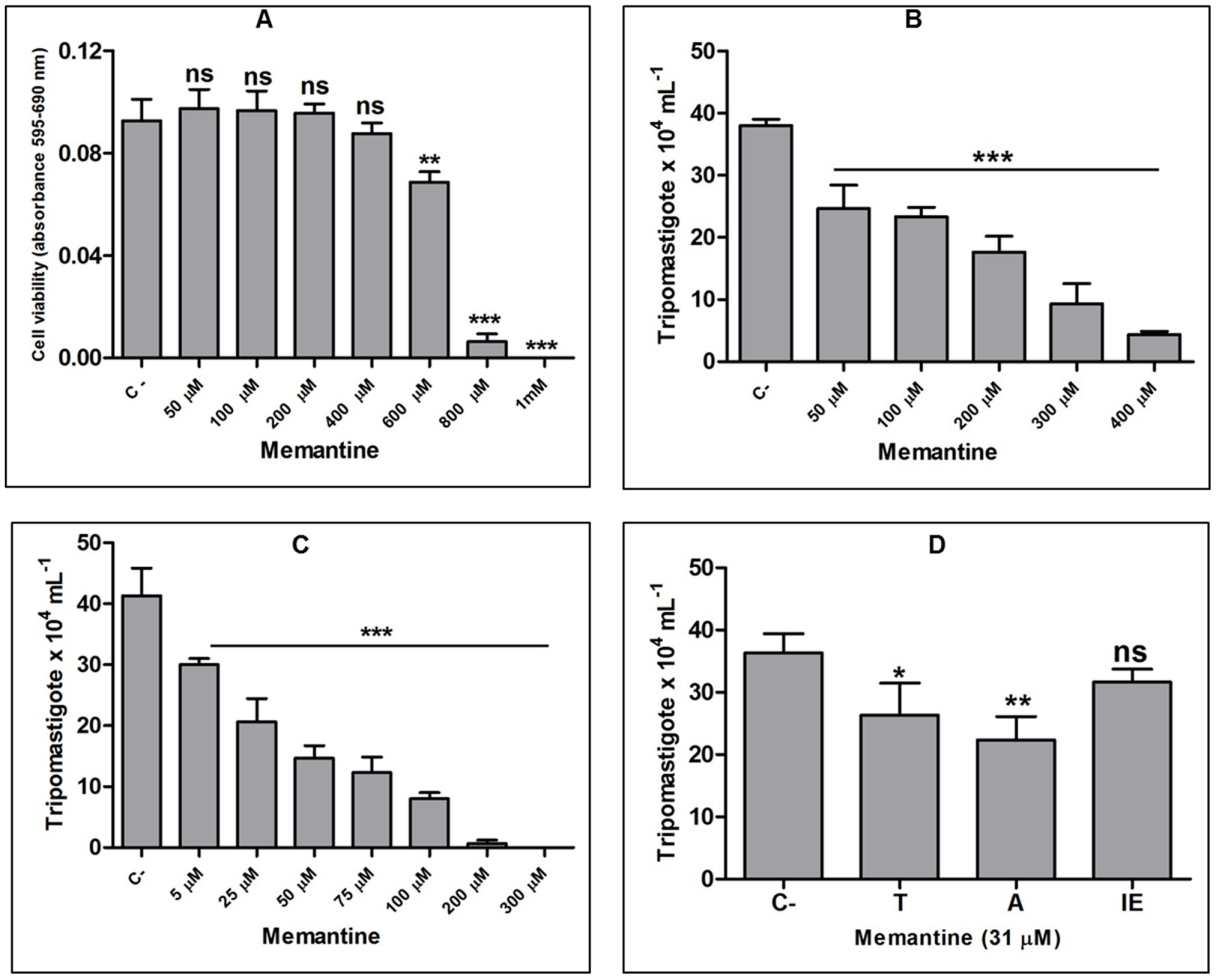
Effect of Memantine on the intracellular cycle of *Trypanosoma cruzi*. Panel A: viability of CHO-K_1_ cells treated with different concentrations of Memantine (range 50 µM to 1 mM). Viability was assessed by MTT assay. Panel B: effect on the infectivity of trypomastigotes treated only during the period of infection (50–400 µM). Panel C: effect of treatment after invasion of parasites in CHO-K_1_ cells (5–300 µM). Panel D: effect of Memantine on intracellular stages. Cells were treated at different stages with 31 µM Memantine (IC_50_ value): **T** (trypomastigote cell invasion), **A** (amastigote) and **IE** (intracellular epimastigote-like) stages. In all experiments, we evaluated the burst of trypomastigotes on the fifth day post-infection by counting parasites in a Neubauer chamber. Tukey test: *: p<0.05; **: p<0.01; ***: p<0.001.

To evaluate the effect of treatment on the intracellular forms of the parasites, we first evaluated the toxicity of Memantine for mammalian CHO-K_1_ cells by MTT assay to avoid cytotoxic doses. Memantine was well tolerated by CHO-K_1_ cells, with an IC_50_ of 624.5±46 µM (Figure 6A). Based on this result, we evaluated the effect of Memantine on parasite infection using concentrations up to 0.4 mM (below the IC_50_ for CHO-K_1_ cells). To verify the effect of the drug on trypomastigote infectivity, CHO-K_1_ cells were infected and treated with different concentrations of Memantine (ranging from 50 to 400 µM) or not treated (control). The parasites were treated for four hours (the interval corresponding to the process of cell invasion). At all concentrations, a significant decrease in the number of trypomastigotes released from the lysed treated cells on the 5^th^ day after infection was observed compared with the control, indicating that Memantine interferes with the infection process, and the IC_50_ under these conditions was determined to be 206.3 µM (Figure 6B). We also evaluated the effect of treatment after invasion of the mammalian cells by *T. cruzi*. All treatments produced a significant reduction in trypomastigote bursting on the 5^th^ day after infection compared with the control (Figure 6C). This result suggests that Memantine also interferes with processes involved in the intracellular cycle. Under these conditions, the IC_50_ value was 31 µM, less than 20 times the IC_50_ for CHO-K_1_ cells (selectivity index: 20.13).

Given the effects of treatment of infected cells throughout the entire infection cycle, we determined which stages of the intracellular cycle (trypomastigote, amastigote or epimastigote-like) are more susceptible to treatment with Memantine. To explore this question, we took advantage of the fact that the CL14 strain produces a synchronic infection in CHO-K_1_ cells as previously reported [17]. In this experiment, 31 µM Memantine (concentration corresponding to the IC_50_ value when applied throughout the infection) was added to the infected cultures at different times: period of infection (four hours), between 24 and 60 hours post-infection (when the parasites are in the host-cells cytoplasm, as amastigotes) and between 60 and 96 hours post-infection (when most of the intracellular parasite population is at the epimastigote-like stage and differentiating into trypomastigotes). The stage most susceptible to treatment was the amastigote stage (Figure 6D), with a 35% decrease in the number of egressed trypomastigotes compared with the control.

## Discussion

The discovery of novel drugs for neglected diseases is a necessity for the development of more efficient chemotherapies. However, some alternative strategies should be followed in parallel to accelerate the process of optimizing the treatment of these diseases. In this sense, the search for new therapeutic uses (in this case, against *T. cruzi*) of well-known drugs already in use for humans (such as Memantine) may help to reduce time- and resource-consuming steps because parameters for their application in humans (such as pharmacokinetics, toxicity, maximum tolerable doses and interactions with other drugs) are already well characterized [10], [26]. Drug repositioning was the main objective of the present work.

The uncompetitive NMDA receptor antagonists Amantadine, Memantine, and MK-801, which are described in the pharmacopeia as antagonists of NMDA glutamate receptors, exhibited trypanocidal activity. These receptors have not been described in *T. cruzi* at the molecular level, although evidence of their existence in *T. cruzi* has been reported [11].

**Figure 7:**
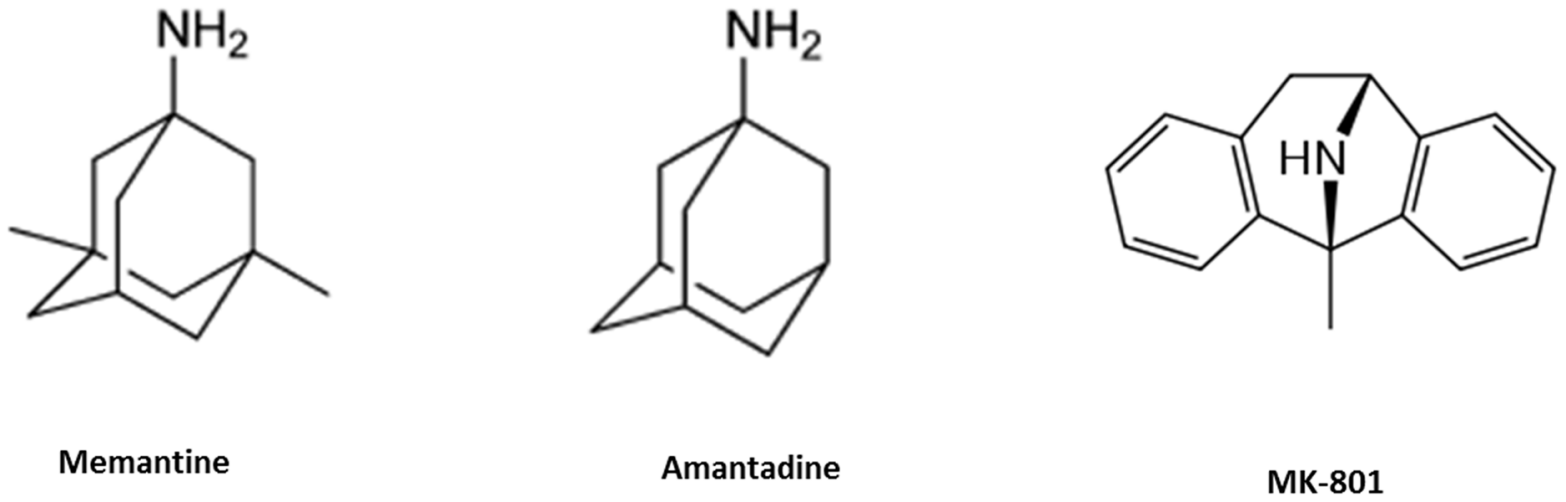
Chemical structures of Memantine, Amantadine and MK-801.

All three evaluated drugs produced a dose-dependent inhibition of proliferation and death in *T. cruzi* epimastigotes. Interestingly, Amantadine and Memantine, which share their basic structure consisting in a tricyclic amine (Figure 7), were the less and the more effective antagonists, respectively. The presence of two methyl groups in Memantine, which are absent in Amantadine, diminished the IC_50_ of the first with respect to the second by a factor of 2.5, showing that little modifications on the leader structure can result in an optimized drug. To investigate the mechanism of death, several parameters were evaluated. First, the integrity of the cytoplasmic membrane and the exposure of phosphatidylserine on the extracellular face were evaluated and strongly suggested PCD with the characteristics of apoptosis. This type of PCD has been described for unicellular protists, including *T. cruzi*, *Leishmania* and *Plasmodium* [27]–[30]. Similar to metazoans, apoptosis is triggered by changes in mitochondrial function. The role of mitochondria in different PCD processes including apoptosis is well characterized [21]–[23]. The production of ROS together with diminished intracellular ATP levels suggest this organelle as a main actor in gating this process. Second, increased intracellular Ca^2+^ levels and morphological changes were consistent with this cell death mechanism. Taken together, these results demonstrate that Memantine triggers PCD with characteristics of apoptosis.

Given that Memantine alters epimastigote physiology, we were interested in determining whether in addition to PCD, this drug may also interfere with differentiation into metacyclic trypomastigotes (metacyclogenesis). This process normally occurs in the terminal midgut of the insect vector. It is worth noting that differentiation requires initial metabolic stress conditions and is mainly energetically supported by amino acids present in reduviid urine and feces, such as proline, aspartate and glutamate [31]. These amino acids allow the parasite to reestablish the intracellular ATP levels required to energize metacyclogenesis [32]. Because Memantine reduces parasite ATP levels, we propose that the inhibition of metacyclogenesis occurs as a result of low ATP levels.

To evaluate Memantine as a trypanocidal of interest for developing new treatments against *T. cruzi* infection, its effect throughout the parasite life cycle in mammalian cells was evaluated. Memantine affected the infectivity of trypomastigote forms, which resulted in a reduced number of trypomastigotes bursted from infected host cells. In addition, the amastigote stage was shown to be the most sensitive stage among those infecting the mammalian cells. This is particularly interesting because amastigotes are the forms involved in maintenance of the chronic phase of infection.

Taken together, these results reveal promising prospects for a new use for Memantine, a drug that is already approved for use in humans, as an anti-*T. cruzi* drug. Preclinical studies are underway to support this proposal.

## Supporting Information

Figure S1 A: Effect of Benznidazole (BZN) on epimastigotes of *T. cruzi* proliferation. **A:** Growth curves of epimastigotes treated with BZN at 28°C and 7.4 pH. black square: 0 µM; black up-pointing triangle: 1 µM; black down-pointing triangle: 10 µM; black left-pointing triangle: 20 µM; black right-pointing triangle: 30 µM; black diamond: 40 µM; black pentagon: 50 µM; black hexagon: 60 µM; black star: 70 µM; black circle: 80 µM; inverse white circle: Inhibition control (0.5 µM antimycin and 60 µM rotenone). **B:** Dose - response curve for BZN.
(TIF)

## References

[1] WHO (2012) Chagas disease (American trypanosomiasis). Available: http://www.who.int/mediacentre/factsheets/fs340/en/. Accessed 15 February 2013.

[2] Almeida-de-Faria M, Freymuller E, Colli W, Alves MJ (1999) *Trypanosoma cruzi:* characterization of an intracellular epimastigote-like form. Exp Parasitol 92: 263–274. 10425154 10.1006/expr.1999.4423

[3] Alves MJ, Colli W (2007) *Trypanosoma cruzi*: adhesion to the host cell and intracellular survival. IUBMB Life 59: 274–279. 17505965 10.1080/15216540701200084

[4] Docampo R (2001) Recent developments in the chemotherapy of Chagas disease. Curr Pharm Des 7: 1157–1164. 11472259 10.2174/1381612013397546

[5] Magdaleno A, Ahn IY, Paes LS, Silber AM (2009) Actions of a proline analogue, L-thiazolidine-4-carboxylic acid (T4C), on *Trypanosoma cruzi* . PLoS One 4: e4534. 19229347 10.1371/journal.pone.0004534 PMC2645137

[6] Rassi A, Marin-Neto JA (2010) Chagas disease. The Lancet 375: 1388–1402. 10.1016/S0140-6736(10)60061-X 20399979

[7] Boscardin SB, Torrecilhas AC, Manarin R, Revelli S, Rey EG, et al (2010) Chagas' disease: an update on immune mechanisms and therapeutic strategies. J Cell Mol Med 14: 1373–1384. 20070438 10.1111/j.1582-4934.2010.01007.x PMC3829005

[8] Urbina JA (2010) Specific chemotherapy of Chagas disease: relevance, current limitations and new approaches. Acta Trop 115: 55–68. 19900395 10.1016/j.actatropica.2009.10.023

[9] Kinnings SL, Liu N, Buchmeier N, Tonge PJ, Xie L, et al (2009) Drug discovery using chemical systems biology: repositioning the safe medicine Comtan to treat multi-drug and extensively drug resistant tuberculosis. PLoS Comput Biol 5: e1000423. 19578428 10.1371/journal.pcbi.1000423 PMC2699117

[10] Nwaka S, Hudson A (2006) Innovative lead discovery strategies for tropical diseases. Nat Rev Drug Discov 5: 941–955. 17080030 10.1038/nrd2144

[11] Paveto C, Pereira C, Espinosa J, Montagna AE, Farber M, et al (1995) The nitric oxide transduction pathway in *Trypanosoma cruzi* . J Biol Chem 270: 16576–16579. 7542649 10.1074/jbc.270.28.16576

[12] Silber AM, Colli W, Ulrich H, Alves MJ, Pereira CA (2005) Amino acid metabolic routes in *Trypanosoma cruzi*: possible therapeutic targets against Chagas' disease. Curr Drug Targets Infect Disord 5: 53–64. 15777198 10.2174/1568005053174636

[13] Duque MD, Camps P, Torres E, Valverde E, Sureda FX, et al (2010) New oxapolycyclic cage amines with NMDA receptor antagonist and trypanocidal activities. Bioorg Med Chem 18: 46–57. 19954985 10.1016/j.bmc.2009.11.017

[14] Lipton SA (2005) The molecular basis of memantine action in Alzheimer's disease and other neurologic disorders: low-affinity, uncompetitive antagonism. Curr Alzheimer Res 2: 155–165. 15974913 10.2174/1567205053585846

[15] de Bartolomeis A, Sarappa C, Buonaguro EF, Marmo F, Eramo A, et al (2013) Different effects of the NMDA receptor antagonists ketamine, MK-801, and memantine on postsynaptic density transcripts and their topography: Role of Homer signaling, and implications for novel antipsychotic and pro-cognitive targets in psychosis. Prog Neuropsychopharmacol Biol Psychiatry 46C: 1–12. 10.1016/j.pnpbp.2013.06.010 23800465

[16] Brener Z, Chiari E (1965) Aspects of early growth of different *Trypanosoma cruzi* strains in culture medium. J Parasitol 51: 922–926. 5848818

[17] Tonelli RR, Silber AM, Almeida-de-Faria M, Hirata IY, Colli W, et al (2004) L-proline is essential for the intracellular differentiation of *Trypanosoma cruzi* . Cell Microbiol 6: 733–741. 15236640 10.1111/j.1462-5822.2004.00397.x

[18] Dolai S, Yadav RK, Pal S, Adak S (2009) Overexpression of mitochondrial *Leishmania major* ascorbate peroxidase enhances tolerance to oxidative stress-induced programmed cell death and protein damage. Eukaryot Cell 8: 1721–1731. 19749178 10.1128/EC.00198-09 PMC2772409

[19] Martins RM, Covarrubias C, Rojas RG, Silber AM, Yoshida N (2009) Use of L-proline and ATP production by *Trypanosoma cruzi* metacyclic forms as requirements for host cell invasion. Infect Immun 77: 3023–3032. 19433547 10.1128/IAI.00138-09 PMC2708565

[20] Mosmann T (1983) Rapid colorimetric assay for cellular growth and survival: application to proliferation and cytotoxicity assays. J Immunol Methods 65: 55–63. 6606682 10.1016/0022-1759(83)90303-4

[21] Irigoin F, Inada NM, Fernandes MP, Piacenza L, Gadelha FR, et al (2009) Mitochondrial calcium overload triggers complement-dependent superoxide-mediated programmed cell death in *Trypanosoma cruzi* . Biochem J 418: 595–604. 19053945 10.1042/BJ20081981

[22] Menna-Barreto RF, Correa JR, Cascabulho CM, Fernandes MC, Pinto AV, et al (2009) Naphthoimidazoles promote different death phenotypes in *Trypanosoma cruzi* . Parasitology 136: 499–510. 19281638 10.1017/S0031182009005745

[23] Smirlis D, Soteriadou K (2011) Trypanosomatid apoptosis: ‘Apoptosis’ without the canonical regulators. Virulence 2: 253–256. 21566464 10.4161/viru.2.3.16278

[24] Kaczanowski S, Sajid M, Reece SE (2011) Evolution of apoptosis-like programmed cell death in unicellular protozoan parasites. Parasit Vectors 4: 44. 21439063 10.1186/1756-3305-4-44 PMC3077326

[25] Gonzales-Perdomo M, Romero P, Goldenberg S (1988) Cyclic AMP and adenylate cyclase activators stimulate *Trypanosoma cruzi* differentiation. Exp Parasitol 66: 205–212. 2840306 10.1016/0014-4894(88)90092-6

[26] Guido RV, Oliva G (2009) Structure-based drug discovery for tropical diseases. Curr Top Med Chem 9: 824–843. 19754397 10.2174/156802609789207064

[27] Al-Olayan EM, Williams GT, Hurd H (2002) Apoptosis in the malaria protozoan, *Plasmodium berghei*: a possible mechanism for limiting intensity of infection in the mosquito. Int J Parasitol 32: 1133–1143. 12117496 10.1016/s0020-7519(02)00087-5

[28] Ameisen JC, Idziorek T, Billaut-Mulot O, Loyens M, Tissier JP, et al (1995) Apoptosis in a unicellular eukaryote (*Trypanosoma cruzi*): implications for the evolutionary origin and role of programmed cell death in the control of cell proliferation, differentiation and survival. Cell Death Differ 2: 285–300. 17180034

[29] Das M, Mukherjee SB, Shaha C (2001) Hydrogen peroxide induces apoptosis-like death in *Leishmania donovani* promastigotes. J Cell Sci 114: 2461–2469. 11559754 10.1242/jcs.114.13.2461

[30] Duszenko M, Figarella K, Macleod ET, Welburn SC (2006) Death of a trypanosome: a selfish altruism. Trends Parasitol 22: 536–542. 16942915 10.1016/j.pt.2006.08.010

[31] Contreras VT, Salles JM, Thomas N, Morel CM, Goldenberg S (1985) In vitro differentiation of *Trypanosoma cruzi* under chemically defined conditions. Mol Biochem Parasitol 16: 315–327. 3903496 10.1016/0166-6851(85)90073-8

[32] Cazzulo JJ (1992) Energy metabolism in *Trypanosoma cruzi* . Subcell Biochem 18: 235–257. 1485353 10.1007/978-1-4899-1651-8_7

