# Supplementary figures and images for: Memantine, an Antagonist of the NMDA Glutamate Receptor, Affects Cell Proliferation, Differentiation and the Intracellular Cycle and Induces Apoptosis in Trypanosoma cruzi
Source: PLoS Negl Trop Dis. 2014 Feb 27;8(2):e2717. doi: 10.1371/journal.pntd.0002717 (PMC3937314; doi:10.1371/journal.pntd.0002717)

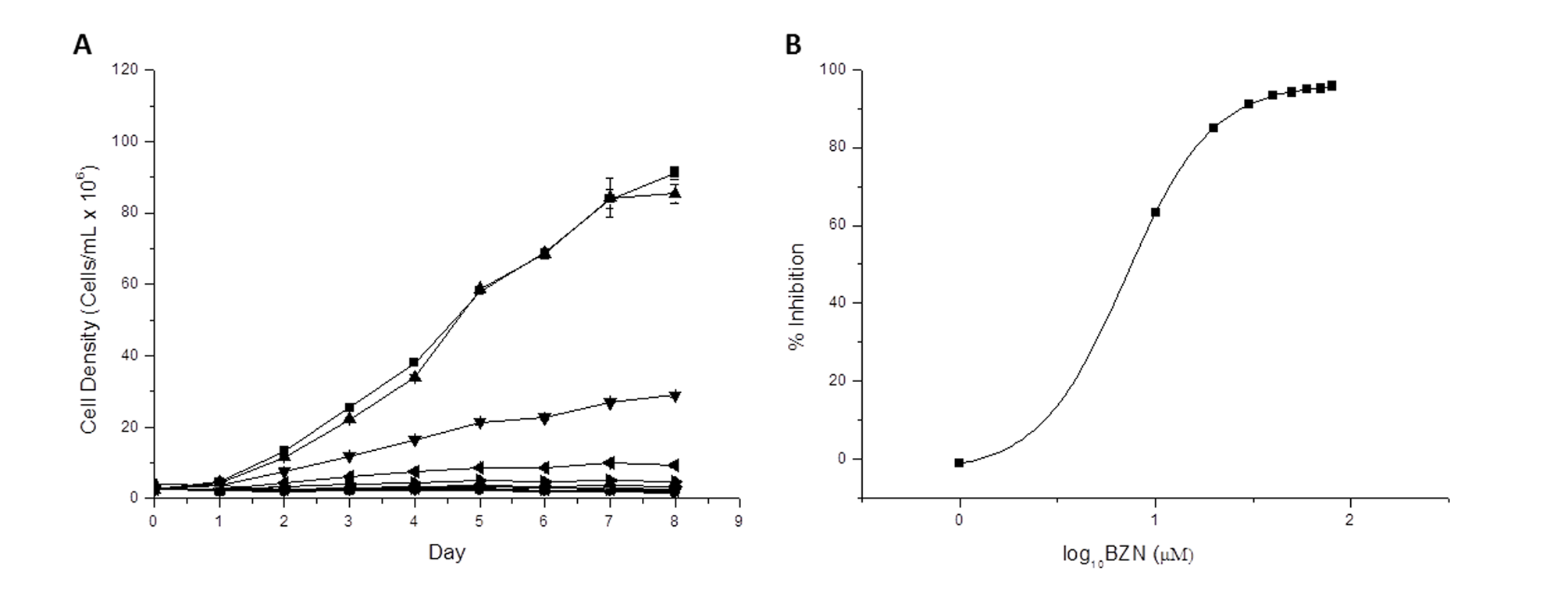

Supplement: Figure S1 — A: Effect of Benznidazole (BZN) on epimastigotes of T. cruzi proliferation. A: Growth curves of epimastigotes treated with BZN at 28°C and 7.4 pH. black square: 0 µM; black up-pointing triangle: 1 µM; black down-pointing triangle: 10 µM; black left-pointing triangle: 20 µM; black right-pointing triangle: 30 µM; black diamond: 40 µM; black pentagon: 50 µM; black hexagon: 60 µM; black star: 70 µM; black circle: 80 µM; inverse white circle: Inhibition control (0.5 µM antimycin and 60 µM rotenone). B: Dose - response curve for BZN. (TIF) [file pntd.0002717.s001.tif]
